# Molecular and epidemiologic analysis of a county-wide outbreak caused by *Salmonella enterica *subsp. *enterica *serovar Enteritidis traced to a bakery

**DOI:** 10.1186/1471-2334-4-48

**Published:** 2004-11-15

**Authors:** Po-Liang Lu, In-Jane Hwang, Ya-Lina Tung, Shang-Jyh Hwang, Chun-Lu Lin, LK Siu

**Affiliations:** 1 Department of Internal Medicine, Kaohsiung Medical University Hospital, 100 Tzyou 1st Rd., Kaohsiung, Taiwan; 2 Infection Control Committee, Kaohsiung Municipal Hsiao-Kang Hospital, 482 Shan-Ming Road, 812 Kaohsiung, Taiwan; 3 Department of Laboratory Medicine, Kaohsiung Municipal Hsiao-Kang Hospital, 482 Shan-Ming Road, Kaohsiung, Taiwan; 4 Division of Clinical Research, National Health Research Institute, 128 Yen-Chiu-Yuan Road, Sec 2, Taipei, Taiwan

## Abstract

**Background:**

An increase in the number of attendees due to acute gastroenteritis and fever was noted at one hospital emergency room in Taiwan over a seven-day period from July to August, 2001. Molecular and epidemiological surveys were performed to trace the possible source of infection.

**Methods:**

An epidemiological investigation was undertaken to determine the cause of the outbreak. Stool and blood samples were collected according to standard protocols per Center for Disease Control, Taiwan. Typing of the Salmonella isolates from stool, blood, and food samples was performed with serotyping, antibiotypes, and pulsed field gel electrophoresis (PFGE) following XbaI restriction enzyme digestion.

**Results:**

Comparison of the number of patients with and without acute gastroenteritis (506 and 4467, respectively) during the six weeks before the outbreak week revealed a significant increase in the number of patients during the outbreak week (162 and 942, respectively) (relative risk (RR): 1.44, 95% confidence interval (CI): 1.22–1.70, P value < 0.001). During the week of the outbreak, 34 of 162 patients with gastroenteritis were positive for Salmonella, and 28 of these 34 cases reported eating the same kind of bread. In total, 28 of 34 patients who ate this bread were positive for salmonella compared to only 6 of 128 people who did not eat this bread (RR: 17.6, 95%CI 7.9–39.0, P < 0.001). These breads were produced by the same bakery and were distributed to six different traditional Chinese markets., *Salmonella enterica *subsp. *enterica *serovar Enteritidis (*S*. Enteritidis) was isolated from the stool samples of 28 of 32 individuals and from a recalled bread sample. All *S*. Enteritidis isolates were of the same antibiogram. PFGE typing revealed that all except two of the clinical isolates and the bread isolates were of the same DNA macrorestriction pattern.

**Conclusions:**

The egg-covered bread contaminated with *S*. Enteritidis was confirmed as the vehicle of infection. Alertness in the emergency room, surveillance by the microbiology laboratory, prompt and thorough investigation to trace the source of outbreaks, and institution of appropriate control measures provide effective control of community outbreaks.

## Background

Salmonellosis, resulting from the ingestion of contaminated poultry, beef, pork, eggs, and milk [1], is an important public health problem worldwide [2]. Although the high temperature of the baking process would suggest that baked goods provide a relatively inhospitable environment for colonization with infectious pathogens, there have been numerous reports of food poisoning outbreaks associated with consumption of baked goods [3-8], and such outbreaks can be a major public health concern [4].

Food-borne disease outbreaks due to *Salmonella *species are relatively uncommon in Taiwan [9] compared to those in the United States [21] and Japan [22]. Thirty-one outbreaks were reported to the Department of Health, Taiwan from 1986 to 1995, which accounted for 5.6% of all outbreaks [9]. Serovar Typhimurium was the leading serovar for *Salmonella *food-borne disease outbreaks but serovar Enteritidis has emerged as a new serovar in Taiwan [23], consistent with similar findings of a worldwide increase in *Salmonella enterica *subsp. *enterica *serovar Enteritidis (*S*. Enteritidis) infections [12,24,25].

We report the findings of the investigation of an outbreak of *S*. Enteritidis that was performed after notification of the rapid increase of the number of patients with febrile gastroenteritis in an emergency room and an unusually high percentage of Group D Salmonella isolated since it comprised only 7.4% of the various Salmonella groups in the hospital one year before the outbreak.

## Methods

### Epidemiological investigation

A rapid increase in the number of attendees due to acute gastroenteritis and fever was noted to have begun on July 28, 2001 at the emergency room (ER) of Kaohsiung Municipal Hsiao-Kang Hospital, and this increase continued for the following six days. Clinical and demographic features and the food reported to have been consumed three days before development of gastrointestinal symptoms for all patients with acute gastroenteritis at the ER from July 28 to August 3, 2001 were reviewed by ER personnel and the infection control team. The infection control team conducted patient interviews and reviewed charts using a standardized case record form. Stool and blood cultures were performed once specimens were available. These specimens were further analyzed by the hospital's clinical microbiology laboratory and the laboratory of the Center for Disease Control, Taiwan (CDC, Taiwan) in Kaohsiung, Taiwan. Charts of all patients with and without acute gastroenteritis who visited the ER from six weeks before until two weeks after July 28, 2001 were reviewed by infection control nurses for the baseline data of the ER. An outbreak-associated case was defined as a patient visiting the ER with the diagnosis of acute gastroenteritis and having *Salmonella *infection. A cohort study of all patients attending the ER with acute gastroenteritis between July 28 and August 3, 2001 was performed in order to test the hypothesis that illness was caused by a specific food.

### Laboratory investigation

#### Surveillance culture

Methods for sample collection, cultivation and isolation were conducted according to the standard protocol of the CDC, Taiwan for food-borne disease outbreaks as described previously [9].

#### Serogrouping and serotyping of Salmonella

*Salmonella *serotypes were determined with the use of antiserum (Difco, Detroit, MI, USA) according to the manufacturer's instructions. Serogrouping and serotyping were performed by the slide agglutination method and tube agglutination method to identify the somatic O antigen and flagellar H antigen, respectively [10].

#### Testing for antimicrobial susceptibility

Antimicrobial susceptibility was determined by the disk diffusion method according to the National Committee for Clinical Laboratory Standards [11]. The antimicrobial agents tested included ampicillin, amoxicillin/clavulanate, gentamycin, cefazolin, cefmetazone, cefperazone, imipenem, ofloxacin, and trimethoprim/sulfamethoxazole. *Escherichia coli *ATCC 25922 was used as the quality control organism.

#### Genomic fingerprinting by pulsed field gel electrophoresis (PFGE)

Total DNA was prepared and PFGE was performed as described previously [12,13]. The restriction enzyme *Xba*I (New England Biolabs, Beverly, MA) was used at the manufacturer's suggested temperature. Restriction fragments were separated by PFGE in 1% agarose gel (Bio-Rad, Hercules, CA.) in 0.5X TBE buffer (45 mM Tris, 45 mM boric acid, 1.0 mM EDTA, pH 8.0) for 25 h at 200 V at a temperature of 14°C, with ramped times of 2 to 40 s using the Bio-Rad CHEF-DRII apparatus (Bio-Rad Laboratories, Richmond, CA). Gels were then stained with ethidium bromide and photographed under ultraviolet light. The resulting genomic-DNA profiles, or "fingerprints," were interpreted according to established guidelines [14]. All experiments above were performed in duplicate.

#### Environmental investigation

Food items having a significant relationship with gastroenteritis cases with *Salmonella *infection were suspected as the vector of infection. A sample of the implicated food was collected from a patient's home within six hours of the patient having symptoms of febrile gastroenteritis and it was stored at 4°C. Further tracing the source of the suspected food and the subsequent environmental investigation were undertaken by the Bureau of Health, Kaohsiung County and CDC, Taiwan.

#### Data collection and statistics

Differences between groups were compared using Chi-Square analysis. Relative risk and 95% confidence intervals were also calculated when Chi-Square analysis was used. Continuous variables were analyzed with Student's *t*-test. Significance was considered at a P value < 0.05 (Epi Info. Version 3.2.2 Centers for Disease Control and Prevention (CDC), USA).

## Results

### Patients

**Figure 1:**
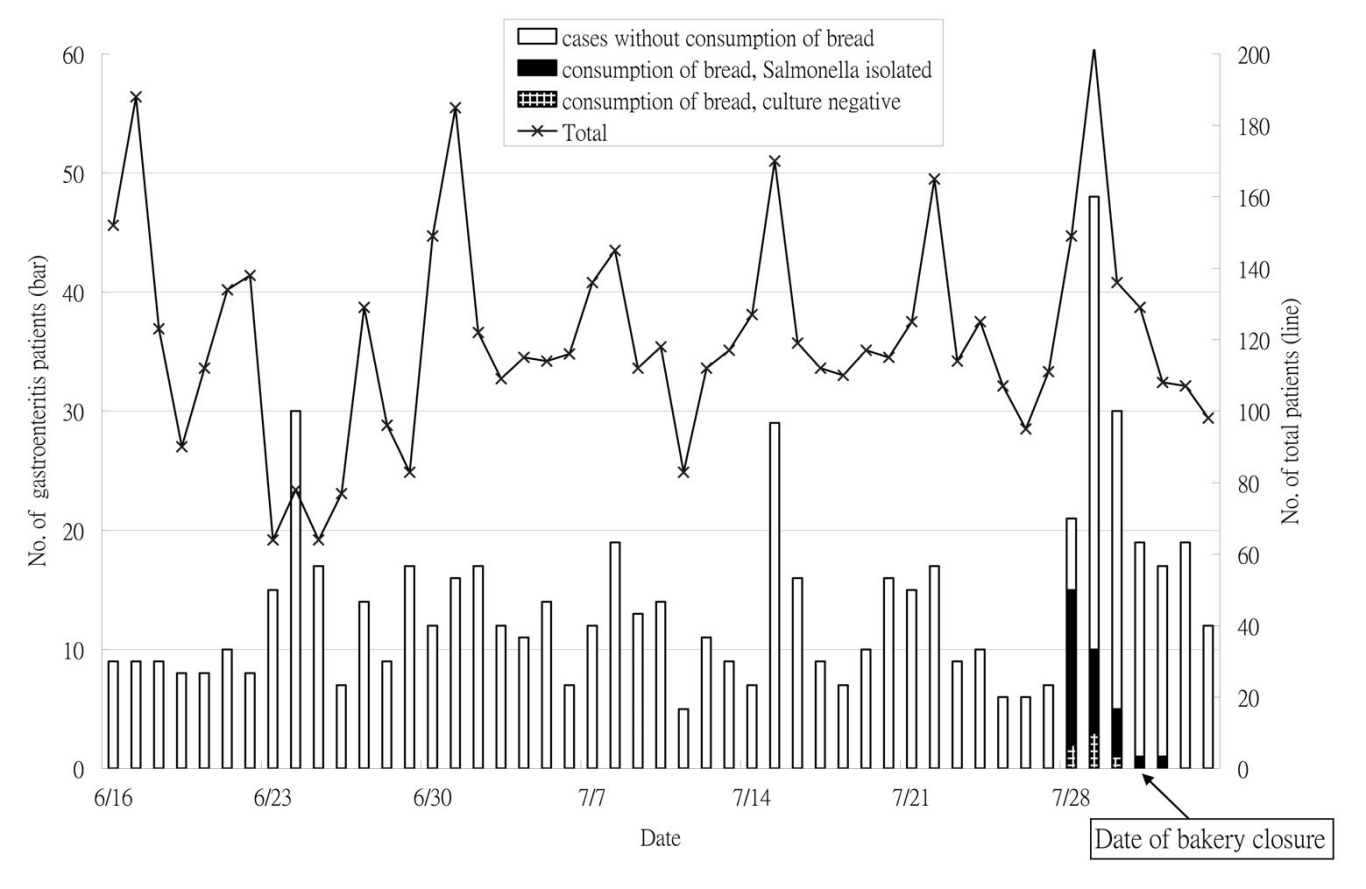
Case number of attendees of the ER due to all causes (line) and due to gastroenteritis (bar) 6 weeks before the outbreak and during the outbreak week. Cases consuming the kind of bread with and without *Salmonella *isolated in late July and early August, 2001 were indicated in different bars.

An increase in the number of patients admitted due to acute gastroenteritis and fever was noticed beginning from the early morning of July 28, 2001 with a return to normal levels one week later. Reviewing daily case-visits to the ER due to all causes and due to gastroenteritis six weeks before this outbreak period revealed significantly more cases due to all causes visiting the ER on Saturday and Sunday than on weekdays (From Monday to Friday) (mean ± standard deviation (S.D.): 140.3 ± 38.3 compared with 109.6 ± 16.7, P = 0.004). The number of patients visiting due to gastroenteritis on Saturday and Sunday were also higher than those visiting on weekdays (mean ± S.D.: 15.8 ± 7.3 compared with 10.5 ± 3.6, P = 0.032) (Fig. 1).

A total of 162 (17.2 %) of 942 patients visiting the ER during the outbreak period had acute gastroenteritis. Compared with 85 (9.7%) of 872 patients in the week before the outbreak, significantly higher percentages of patients with gastroenteritis were observed in the outbreak week (Relative risk (RR): 1.92, 95% confidence interval (CI): 1.38 – 2.26, P < 0.001). A comparison of the number of patients with and without acute gastroenteritis (506 and 4467, respectively) during the six weeks before the outbreak week and during it also revealed a significant increase in the number of patients of gastroenteritis during the outbreak week (RR: 1.44, 95% CI: 1.22–1.70, P value < 0.001).

Investigation of food consumed by gastroenteritis patients during the outbreak period revealed 34 (21%) had consumed the same kind of egg covered bread decorated with mayonnaise and fried seasoned pork fiber from six traditional Chinese markets (three located in Kaohsiung City and three in Kaohsiung County). There were 2, 2, 2, 3, 3, and 22 gastroenteritis patients that consumed the bread purchased from 6 markets, respectively. In total, 28 of 34 patients who ate this bread were positive for *Salmonella *compared to only 6 of 128 people who did not eat this bread (RR: 17.6, 95%CI 7.9–39.0, P < 0.001). The association of consuming the kind of bread and having *Salmonella *infection was significant (RR: 17.6, 95% CI: 7.9–39.0, P value < 0.001). No other identified food or restaurant exposure was significantly associated with the outbreak.

Regarding the 28 *Salmonella *cases that consumed the implicated bread, 12 were male. Their age ranged from three to 71 years old. Eleven cases (39.3%) were under 18 years old and one case was over 65. The incubation periods ranged from 4 to 17 (median, mean ± S.D. 10, 9.4 ± 3.2) hours after consumption of the bread. Twenty-seven of the 28 patients were hospitalized. The clinical symptoms among the 28 cases included abdominal pain (100%), fever (100%), diarrhea (100%), vomiting (85.7%), chills (35.7%), and headache (7.1%). The laboratory data showed eight (28.6%) cases had white blood cell counts higher than 10000 /mm^3^. Positive occult blood reaction was found in stool specimens of 24 of 27 (88.9%) patients tested. Colitis was found in all four patients who received colonoscopy examination. The two bacteremia patients' fever subsided one day and two days after admission, respectively, without antimicrobial therapy. No mortality or sequellae occurred among these cases during hospitalization or in the three months' follow-up by infection control nurses.

Further investigation by the city public health administration found the incriminated bread from the six markets was all produced by the same bakery that was prohibited from production of all kinds of bread on August 1, 2001. Because the bakery had stopped their production one day before the official prohibition, no further samples of the components of bread, such as the egg and mayonnaise, were available. Investigation of the bakery staff with stool cultures for *Salmonella *and surveillance culture of the workplace surfaces were not performed.

### Bacterial strains

In the week of this outbreak, there was an extraordinarily high percentage (94.1%) of group D of *Salmonella *isolates. Twenty-eight *Salmonella *isolates were cultured from 32 available stool specimens from 34 patients who consumed the implicated food. These isolates were all of group D and were further identified as *S*. Enteritidis. This pathogen grew in two of the 34 patients' blood cultures. A *Salmonella *isolate of the same serovar was cultured from bread provided by a patient. The sample had been stored in the refrigerator because the patient had not finished eating it.

A total of 30 stool specimens from 25 patients not consuming the implicated bread were collected and *Salmonella *was isolated from six of the 25 patients' stool specimens. For the six *Salmonella *isolates from cases not consuming the implicated food during the outbreak period, two were of group B and four were of group D. Further serovar analysis and PFGE analysis of the four group D isolates was not performed. Among the four group D isolates, two were resistant to ampicillin and trimethoprim/sulfamethoxazole, which was different to the antibiogram of the isolates from cases consuming the implicated bread.

### Antibiogram and PFGE patterns

**Figure 2:**
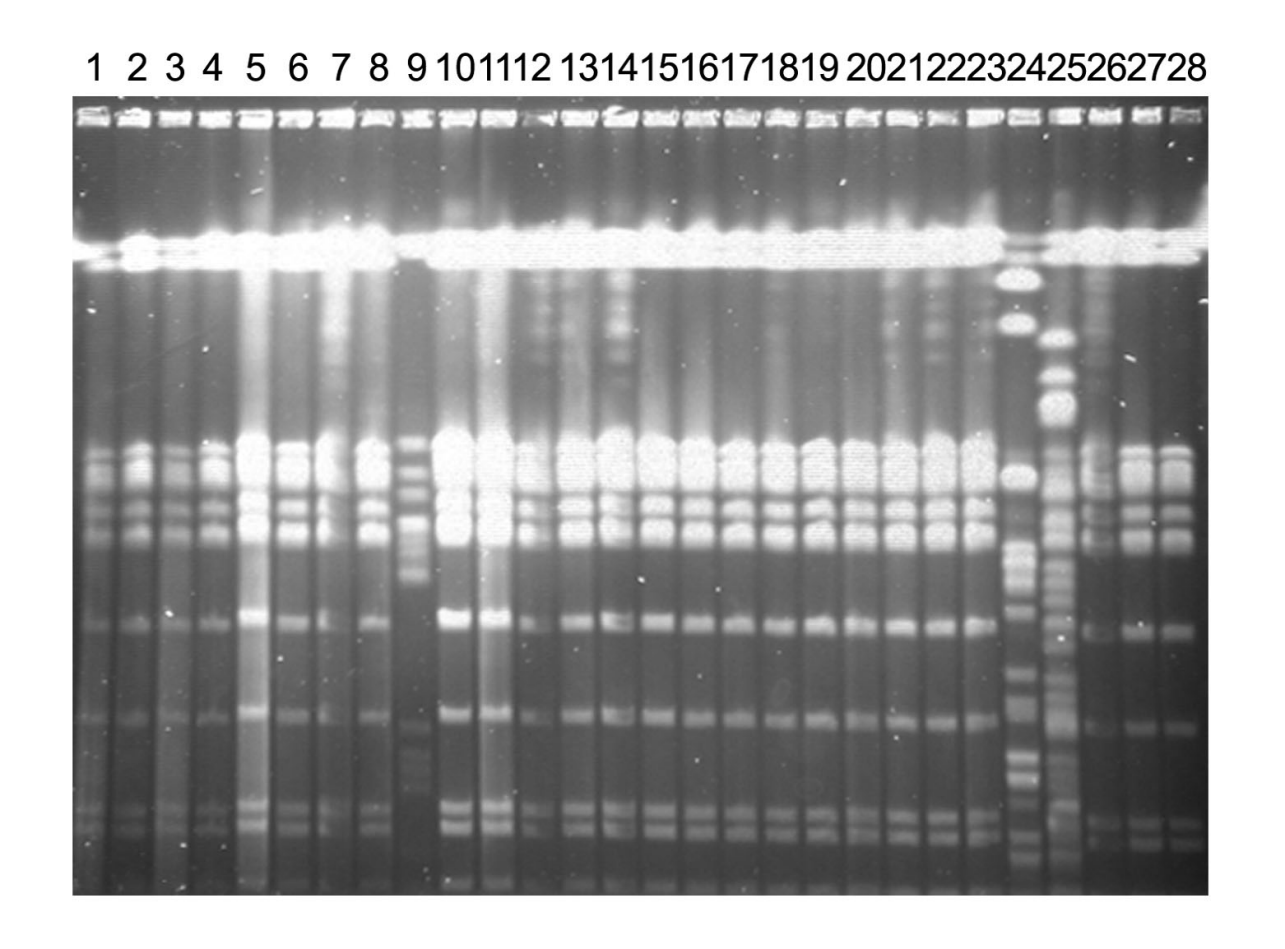
Agarose gels of the PFGE profiles of *Salmonella *Enteritidis isolates. Lane 1–28: *Salmonella *DNA digested with restriction enzyme *Xba*I. Except for lane 9 which is the macrorestriction pattern from a outbreak-unrelated strain during the outbreak period from a patient not consuming the index food (bread), all the lanes were from outbreak-related isolates. Lane 24 and lane 25 were from two cases consuming bread from two different markets which showed the only two different PFGE patterns to other outbreak-related isolates having the same PFGE pattern. Lane 20 was from the strain isolated from bread. Lane 21 is from a blood isolate and lane 22 was from a stool isolates of the same case and the two lanes were of the same PFGE pattern.

All 28 stool isolates, two blood isolates and one food isolate were susceptible to the nine antimicrobial agents tested. Only two stool isolates showed unrelated PFGE patterns with more than six band differences compared to the epidemic PFGE pattern of the other isolates. All the other stool, blood, and food isolates had the same PFGE pattern indicating a clonal relationship (Figure 2). The two isolates with different PFGE patterns were from two patients who had purchased and consumed the bread from two different markets.

## Discussion

The source of the outbreak was traced to ingestion of egg-covered bread topped with mayonnaise and fried seasoned pork fiber from six traditional Chinese markets, and then back to a bakery. This outbreak had no clear association with the common sources of food poisoning outbreaks in Taiwan, such as commercial lunch boxes, or food from banquets or wedding dinners, and it was caused by an uncommon pathogen of food-borne outbreak in Taiwan [9].

Most of the patients falling ill with gastroenteritis on the 29th and 30th of July (Saturday and Sunday) had not consumed the implicated bread, (Fig. 1) suggesting some other source or vehicle existed. However, it could be due to the typical increase of ER visits on Saturdays and Sundays, compared to weekdays, because most clinics stop services on weekend.

Compared with phage typing [15] and plasmid typing [12,16,17], pulsed-field gel electrophoresis (PFGE) is a reproducible, discriminative, and feasible typing method for *S*. Enteritidis [18,19] though it has been suggested to have limited value in epidemiological analysis because of the high genetic homogeneity among strains of *S*. Enteritidis [20]. Analytic epidemiologic study in addition to molecular typing is essential in thoroughly investigating the source of *Salmonella *isolates.

Outbreaks can be missed easily if the increase in case number is not noticed due to the wide distribution of outbreak sources in different areas or if a relatively small outbreak occurs among a large population and cases are exposed to the outbreak source at different times. Recognition of the outbreak reported in this study was aided by the routine surveillance of *Salmonella *groups and knowledge that the percentage of group D *Salmonella *patients was low before. Thus, the unusually high percentage of group D *Salmonella *seen within the week of the outbreak led to the investigation. The implicated vehicle of infection in the outbreak was an egg-covered bun topped with mayonnaise and fried seasoned pork fiber which had been distributed to different markets after production at the same bakery. Epidemiological study, antibiograms, and serologic and molecular typing patterns revealed that almost all cases of *S*. Enteritidis infection during the period were the same as that of the bread isolate. Only the implicated baked good from the same bakery caused *Salmonella *infections while other items from the bakery were not found to be epidemiologically related with the outbreak. This finding suggests contamination of the pathogen did not occur during the common routes of production, transportation, or selling of goods from the bakery. Although the bread sold in the traditional Chinese markets frequently does not conform to sanitary requirements, and although the staff involved in distribution and selling are not subject to the routine hygiene inspections, in food stores, the possibility of contamination of a specific bakery product simultaneously at six markets in the same time period is very low, suggesting that the contamination occurred during the production process. Food contamination in the outbreak was traced to the same bakery but an investigation of infection among the bakery staff and sources of the contents of the bread was not conducted. Although the baking process involves high temperatures sufficient to kill pathogens, the manual addition of toppings or flavors, such as mayonnaise, eggs, and meat products provide possibilities for contamination with food-borne pathogens. In addition, insufficient baking may be a risk factor for human health because it may not destroy microbial contamination.

Different *Salmonella *serovars have been related specifically with some foods. *S*. Enteritidis is particularly related to eggs [26-28]. In this outbreak and the other *S*. Enteritidis outbreaks related to baked goods[4,5,7,8], all of the food products contained eggs and the egg material had not been cooked sufficiently. Whether the topping of this bread with lightly cooked eggs or the under-cooking played a role in the contamination could not be confirmed due to lack of culture of separate parts of the bread. The finding that *Salmonella *isolated from the bread had the same serovar, antibiogram, and PFGE pattern as isolates from patients and the significant relationship between consumption of this baked good and the isolation of *Salmonella *implicated this food product as the vehicle of contamination.

This outbreak had a relatively short incubation compared to the 24–72 hour range reported for other *Salmonella *outbreaks [29,30]. Whether a highly virulent strain or a high inoculum of bacteria [30] during production or rapid growth of bacteria due to hot summer weather, contributed to the short incubation time was not investigated.

For many food-borne outbreaks, the pathogens and transmission vehicles are often not identified, usually because of delayed collection of epidemiologic and microbiologic information [28]. Initiating an outbreak investigation based on surveillance of emergency room admissions would provide useful information which may lead to early recognition of the pathogen and vehicle [31]. The alertness of our emergency room staff resulted in recognition of the unusual increase in cases of gastroenteritis, although the patients came from two districts and had no obvious relationship to common food sources. This led to prompt investigation and containment of a potential source of further infection. Cooperation of the emergency room, microbiology laboratory, the infection control team staff at hospitals and public health administration staff combined with the application of epidemiological and bacterial typing methods is crucial to the success of the source identification to prevent further dissemination during *Salmonella *Enteritidis outbreaks.

## Competing interest

The author(s) declare that they have no competing interest.

## Authors' contributions

Po-Liang Lu and L. K. Siu were in charge of the investigation, data handling and writing of the manuscript. Po-Liang Lu and Shang- Jyh Hwang participated in the design of the study. Po-Liang Lu, In-Jane Hwang, Ya-Lina Tung and Shang-Jyh Hwang were in the investigation team for data collecting and data analysis. Po-Liang Lu performed the statistical analysis. Chun-Lu Lin and L. K. Siu carried out microbiological assays. All authors have read and approved the final manuscript.

## Pre-publication history

The pre-publication history for this paper can be accessed here:
